# An Analysis of Three *Pistacia* Species’ Phenolic Compounds and Their Potential Anticancer and Cytotoxic Activities on Cancer Cells—A Review

**DOI:** 10.3390/cimb47060393

**Published:** 2025-05-26

**Authors:** Naser A. Alsharairi

**Affiliations:** Heart, Mind and Body Research Group, Griffith University, Gold Coast, QLD 4222, Australia; naser.alsharairi@gmail.com

**Keywords:** *Pistacia vera*, *Pistacia terebinthus*, *Pistacia khinjuk*, phenolic compounds, cytotoxicity, anticancer activities

## Abstract

The genus *Pistacia* from the Anacardiaceae family contains species of wild flowering plants. The only species that produces edible nuts large enough for commercial sale is *P. vera* L. (pistachio). Other species, such as *P. terebinthus* L., *P. atlantica* L., and *P. khinjuk*, are used as pistachio rootstocks. *Pistacia* species include phenolic compounds, such as flavonoids, essential oils, and tannins, which are responsible for a number of pharmacological properties. The species most commonly investigated for their anticancer and/or cytotoxic activities against cancer cells in experimental studies include *P. lentiscus*, *P. atlantica* subspecies, and *P. chinensis* subsp. *integerrima*. However, no review exists that evaluates the phenolic compounds of three other *Pistacia* species (*P. vera* L., *P. terebinthus* L., and *P. khinjuk*) and their anticancer and cytotoxic effects. Thus, this review aims to thoroughly assess the phenolic compounds that were isolated from these species and investigate any potential anticancer or cytotoxic effects on cancer cells. The findings show that *pistacia* species and their isolated phenolic compounds (phenolic acids, flavonoids, and essential oils) from different plant parts have anticancer activity against lung, cervical, prostate, gastric, colon, liver, renal, skin, and breast cancer cells. Additionally, certain phenolic compounds from *pistacia* species have cytotoxic activity; however, the degree of toxicity may vary based on the dosage and duration of use. Further experiments are required to fully understand the possible mechanisms underlying the anticancer and cytotoxic effects of *pistacia* species and their phenolic compounds on cancer cells.

## 1. Introduction

The genus *Pistacia* comprises wild flowering plant species in the Anacardiaceae family that are distributed across Asia, Europe, Africa, North America, and the Middle East [1]. The most common species of *Pistacia* are *P. vera* L. (pistachio nuts), *P. lentiscus* L. (mastic gum), *P. terebinthus* L. (terebinth), *P. atlantica* L. (baneh), *P. chinensis* L. (Chinese pistachio), and *P. khinjuk* Stocks [2,3]. *P. vera* L. is the only species that yields edible nuts large enough to be sold commercially, while other species are utilized as rootstocks for pistachios or in agroforestry ecosystems [4,5].

The pistachio tree (*P. vera* L.) is mostly deciduous and dioecious, and grows well in arid areas with low soil [5,6,7]. The pistachio’s fruits have solitary and oblong seeds that are coated in a soft layer called testa. The endocarp is a cream-colored shell that covers the seeds, which is then covered by a fleshy hull that is pale green with a red flush [6]. The seed is comprised of two cotyledons and ranges in color from light to dark green or greenish yellow [6]. The distinctive color of pistachios is due to a mixture of bioactive compounds (e.g., lutein, zeaxanthin, and catechins give pistachios their yellow hue) [6]. Pistachio nuts are rich in mono-and polyunsaturated fatty acids, fiber, amino acids, vitamins, and minerals. Pistachios are also a good source of a variety of bioactive compounds, including carotenoids, anthocyanins, proanthocyanidins, flavones, flavanols, flavanones, and isoflavones [6,8,9]. Several pharmacological activities, such as antioxidant, anti-inflammatory, cardioprotective, antihypertensive, antidiabetic, and analgesic/antipyretic effects, have been reported for pistachio nuts both in vitro and in vivo [2,3,6,9,10,11].

*P. terebinthus* L., also referred to terebinth or the turpentine tree, is a dioecious shrub native to Greece, Portugal, Turkey, the Canary Islands, and the Middle East [11]. This species thrives in moist areas, in pine forests, and on rocky, particularly calcareous, slopes [12]. Because *P. terebinthus* subsp. *palaestina* (Boiss.) Engler and *P. terebinthus* subsp. *terebinthus* share a lot of morphological similarities, they are classified under the *P. terebinthus* L. species [13]. Terebinth is considered a natural food additive used in the making of ice cream, due to its high mineral contents, organic acids, and appealing color/flavor qualities [14]. The resin extracted from *P. terebinthus* L. aids in the immobilization of yeast cells, aiming to produce sucrose, glucose, and maltose at different temperatures through alcoholic fermentation [15]. The terebinthus kernel differs from the skin because it has higher levels of γ-tocopherol, polyunsaturated fatty acids, and total sterols. In contrast, the terebinthus skin has a higher content of α-tocopherol and saturated fatty acids than the kernel; therefore, it may be used in the cosmetics sector [16]. *P. terebinthus* L. is utilized to cure medical conditions, such as diuretic and urinary inflammations [11]. It also has anticholinesterase, antioxidant, antimicrobial/antibacterial, neuroprotective, and antidiabetic properties [17,18,19,20,21,22]. *P. terebinthus* L. produces a diverse array of bioactive compounds, such as flavonoids, tannins, organic acids, essential oils, and resin [11].

*Pistacia khinjuk* Stoks (*P. khinjuk*) is a resinous dioecious tree with petalless flowers and opposite pinnate leaves with exstipulation that grows in Turkey, Iran, India, Afghanistan, Pakistan, and the Middle East [23]. *P. khinjuk* demonstrated a high nutritional value, which is explained by its high levels of fatty acids, tocopherols, tocoterienols, protein, vitamin C, calcium, copper, iron, and potassium [24,25,26]. *P. khinjuk* has been thoroughly studied for a range of pharmacological properties, which have been shown to exert antioxidant, anti-inflammatory, antibacterial/antimicrobial, antileishmanial, and antihyperlipidemic activities [20,24,27,28,29,30,31,32,33,34,35,36].

In experimental investigations, *P. lentiscus*, *P. atlantica* subspecies, and *P. chinensis* subsp. *integerrima* are the species most extensively reviewed for their anticancer and/or cytotoxic activities against cancer cells. The myricetin found in *P. lentiscus* extracts has anticancer activities against breast and melanoma cancer cells and may also have strong cytotoxic effects on melanoma cancer cells [37]. *P. lentiscus* var. chia gum has been demonstrated to inhibit the growth and induce apoptosis in prostate, colorectal, leukemia, and melanoma cancer cells [38]. With minimal cytotoxic effects on cancer cells, it has been reported that the mastic gum resin from *P. atlantica* subsp. *kurdica* and the ethanolic extract from *P. atlantica* Desf. leaves inhibits the growth of gastric carcinoma and cervical cancer cells, and causes apoptosis and cell cycle arrest in lung and prostate cancer cells [39]. The alkaloids, tannins, and sterols found in *P. chinensis* subsp. *Integerrima* extracts have anticancer activities but also may have strong cytotoxic effects on breast cancer cells [40].

The phenolic compounds and the cytotoxic and anticancer activities of *P. vera* L., *P. terebinthus* L., and *P. khinjuk* have not yet been specifically reviewed. Consequently, in this review, a thorough evaluation of the phenolic compounds isolated from these species was conducted, and any potential cytotoxic or anticancer effects on cancer cells were investigated.

## 2. Methods

A literature search was performed using a combination of terms, including “*P. vera*”, “*P. terebinthus*”, “*P. khinjuk*”, “phenolic compounds”, “cytotoxicity”, and “anticancer activity”, in PubMed/Medline and Scopus databases. All English-language articles published up until the end of March 2025 that addressed these species and provided clear evaluations of their anticancer and cytotoxic activities on cancer cells were included. In total, 59 studies were included after the full texts and titles were screened.

## 3. Phenolic Compounds of *Pistacia* Species

Phenolic compounds are a diverse class of secondary metabolites produced by the metabolism of plants [41]. Phenolic compounds can be classified as either flavonoids or non-flavonoids [41]. Depending on their degree of hydrogenation, flavonoids can be classified as flavanones, flavones, flavonols, isoflavones, and anthocyanins [42]. Phenolic acids are among the most typical non-flavonoid compounds [43]. The flavonoid and non-flavonoid compounds that have been extracted from *P. vera* L., *P. terebinthus* L., and *P. khinjuk* are presented in this section.

### 3.1. Pistacia Vera

The ripe fruits of five different *P. vera* L. cultivars were found to contain different amounts of total phenols, flavonoids, and proantocyanidins across four different Iranian regions [44]. A higher extraction yield was reported with fewer polar solvents when three different types of solvents were used to make the hull and kernel extract of *P. vera var*. Sarakhs in Iran. The highest concentrations of total phenols, flavonoids, and proanthocyanidins were found in the ethanolic hull and kernel extract when compared to ultrapure water, ethyl acetate, hexanea, and methanol extracts [45]. The main phenolic compounds that were isolated from *P. vera* L. cultivars in Iran were gallic acid, catechin, eriodictyol-7-*O*-glucoside, and cyanidin-3-*O*-galactoside. The cultivars of Kaleghouchi and Fandoghi were found to have the highest total flavonol and phenolic contents among genotypes [46]. Two pistachio cultivars, Kalleghuchi and Ohadi, were extracted in Iran using four different solvents and ultrasonic/maceration, and the results revealed a difference in the content of total phenolics, flavonoids, and anthocyanins [47]. The pectinase-treated extract permeate of Iranian-produced pistachio green hull may have an impact on increasing total phenols and flavonoids [48].

The capability of subcritical water extraction for the recovery of dried ground pistachio hull phenolic compounds was evaluated in Turkey in contrast to an ultrasound-assisted procedure employing aqueous methanol. The subcritical water extracts showed larger yields of total flavonol and gallic acid derivatives than the aqueous methanol extracts when the extraction temperature was increased from 110 to 170 °C [49]. The phytochemical composition of pistachio hull ethanolic and aqueous extracts made via microwave-assisted extraction was examined in one study conducted in Turkey. The findings demonstrated that the pistachio hull had large levels of quercetin, gallic acid, and total phenolic and flavonoid contents [50]. The phenolic compounds of *P. vera* L., which are native to Greece/Turkey, were examined utilizing two microwave-assisted extraction techniques in conjunction with high-pressure liquid chromatography. The findings showed that gallic acid had the greatest quantities of all the compounds [51]. One study conducted in Tunisia examined the extraction of phytochemicals from fresh *P. vera* L. leaves. In contrast to maceration extraction, microwave-assisted extraction produced a greater extraction yield of total phenolic and flavonoid contents [52].

Phytochemical and bioactivity investigation of fresh raw un-hulled pistachios harvested in California revealed that the main compounds were anacardic acids, carotenoids, phytosterols, fatty acids, and quercetin-3-*O*-glucoside. Quercetin, myricetin, and luteolin were found in smaller amounts [53]. Under high temperature (220 °C) and pressure (6.5 MPa), several flavonoids and derivatives of gallic acid were identified in a study that aimed to optimize the subcritical fluid extraction method using either pure water or ethanol to extract phenolic compounds from defatted pistachio nuts grown in Argentina [54]. Another study reported that the acidified methanolic extracts of *P. vera* cv Kerman cultivars from Argentina included a number of phenolic components that were detected and quantified [55]. During the pistachio oil production process in Argentina, a study was conducted to evaluate the phenolic compounds present in both the oil and the flour. Pistachio flour exhibited significant levels of catechin, gallic acid, and cyanidin-3-*O*-galactoside, whereas pistachio oil showed high levels of oleic and linoleic acid, chlorophylls, and carotenoids [56].

The hydrophilic extract of the Sicilian Bronte’s cultivar of *P. vera* L. nuts included a considerable quantity of proanthocyanidins, trans-resveratrol, and the isoflavones genistein and daidzein [57]. Several extraction methods were applied in a study on dried *P. vera* L. shells grown in the Sicilian district of Bronte. The highest concentration of flavonoids and phenols was obtained using the methanol extraction method [58]. According to a different study on the phenolic components of Bronte pistachios, the levels of phenolic compounds in the pistachio skins and seeds varied [59]. A study into the differences in the phenolic profiles of eight *P. vera* L. cultivars and their virgin oils cultivated in Spain was conducted. Variations across cultivars were found, with the Larnaka variety having a noticeably greater phenolic content. The flavanols, flavonols, flavanones, anthocyanins, and gallotannins were the main phenolic groups found in pistachio nuts. Virgin pistachio oils had a very low phenolic content, and the only compound detected was eriodyctiol [60]. A study was carried out to describe eleven distinct varieties of pistachio kernels that were gathered in 2019 and 2020 from Andalusia, Spain. The cultivars of pistachios with the highest levels of total polyphenols were Larnaka, Avdat, Aegina, and Mateur. The most abundant polyphenolic group was found to be hydroxybenzoic acids, which were followed by flavanols [61].

The major phenolic compounds found in *P. vera* L. are summarized in Table 1.

### 3.2. Pistacia Terebinthus

Most studies examining the phenolic compounds of *P. terebinthus* L. were carried out in Turkey. In one study, oleic, palmitic, and linoleic acids were identified as the primary fatty acids in *P. terebinthus* L. fruits [20]. Leaves of *P. palaestina* are shown to be rich in essential oils, particularly α-pinene and sabinene [21]. It was reported that the terebinth oil’s fatty acid content varied, with oleic acid being the most abundant [62]. *P. terebinthus* seeds were found to be abundant in α- and γ-tocopherol, β-sitosterol, linoleic acid, palmitic acid, and oleic acid [63]. Terebinth fruits were rich in minerals, as well as fatty acids, such as 9-hexadecenoic, palmitic, and linoleic acids [64]. Phenolic compounds were found in considerable amounts in bread made by adding turpentine fruit paste in different concentrations [65]. *P. terebinthus* L. fruits were found to contain increased levels of phenolic compounds (quercetin and catechin) and fatty acids (palmitic, oleic, and linoleic) after 30 min of pre-sonication [66]. Oils extracted from *P. terebinthus* fruits roasted at 180 °C for 0–40 min were shown to have low levels of β-carotene and lutein, but a high total phenolic content, assessed in gallic acid equivalent [67]. It was found that methanol extract made from *P. terebinthus* L. subsp. *terebinthus* has more flavonoids and phenolics than acetone extract [68]. The methanol extract of *P. terebinthus* fruits was found to contain the greatest extracts in total phenols, while the ethyl acetate extract was found to contain the highest extracts in total flavonoids. The findings also indicated that the main compounds of the fatty and essential oil of *P. terebinthus* fruits were oleic acid and α-pinene [69]. Compared to the other extracts, the acetone extract of fresh *P. palaestina* leaves grown in Syria at a 40% concentration had the highest total phenolic content (expressed in gallic acid equivalents) [22].

An overview of the major phenolic compounds found in *P. terebinthus* L. is shown in Table 2.

### 3.3. Pistacia Khinjuk

The highest concentration of the three fatty acid compounds that were extracted from *P. khinjuk* seeds cultivated in Turkey was found to be that of oleic acid [20]. The fresh fruits of *P. khinjuk*, which grows in Iran, were found to contain several compounds in their essential oil, with phellandrene being the most abundant [70]. The major fatty acid found in the hull and kernel of *P. khinjuk* cultivated in Iran was oleic acid, while other fatty acids were also extracted [24]. Fatty acid compounds were found in high concentrations in the hull and kernel of ripe *P. khinjuk* fruits in three Iranian regions. In contrast, the hull contained higher concentrations of phenolic compounds than the kernel [25]. The oil extracted from fresh leaves of *P. khinjuk* cultivated in Iran revealed diverse compounds, with the major one being myrcene [71]. The phenolic profile of different parts of the Iranian-grown *P. khinjuk* fruit revealed that the hull extract had a higher total phenolic content than the kernel and shell [27]. The highest concentration of β-caryophyllene was found in the 56 compounds extracted from the hull essential oil of *P. khinjuk* that was gathered from Iran [28]. In Pakistan, the fresh fruits of *P. khinjuk* showed high levels of flavonoids and phenols when extracted with various solvents [29].

A list of the major phenolic compounds found in *P. khinjuk* is shown in Table 3.

## 4. Anticancer and Cytotoxic Activities of *Pistacia vera*

According to several experimental studies, *P. vera* L. extracts and isolated compounds were evaluated for their anticancer and cytotoxic activities against various cancer types. Using the MTT [3-(4,5-dimethylthiazol-2-yl)-2,5-diphenyltetrazolium bromide] assay, an in vitro experiment showed that 46 essential oil compounds from *P. vera* L. had a dose-dependent cytotoxic effect on breast, ovarian, and colon cancer cells, with half-maximal inhibitory concentration (IC_50_) values typically less than 500 µg/mL [72]. In human hepatoblastoma HepG2 cell lines, the pistachio kernel’s cytotoxic activity against aflatoxin B1 was assessed. Pistachio extract at varying doses (0–60 μM) was applied to cells before, during, and following aflatoxin B1 treatment. The findings demonstrated that the pistachio extract enhanced protein 53 (p53) expression and caspase-3 activity in HepG2 cells, and that it only made aflatoxin B1 more lethal when applied prior to treatment [73]. In a different experiment, *P. vera* L. aqueous extracts at varying doses (18.5, 37, 65, 125, 250, 500, and 1000 µg/mL) did not exhibit in vitro cytotoxic effects on HepG2 cells [74]. An in vitro experiment was performed to evaluate the effects of a methanolic extract from *P. vera* L. hulls on melanoma cell cytotoxicity, cellular tyrosinase activity, and melanin levels. The findings showed that, in comparison to butylated hydroxytoluene and kojic acid (tyrosinase inhibitor), *P. vera* L. hulls had modest anti-tyrosinase activity and a valuable 1,1-diphenyl-2-picryl-hydrazyl radical scavenging effect. Additionally, melanoma cells showed strong cytotoxic and anti-melanogenic effects when the extracts were given at a high concentration (0.5 mg/mL), which diminishes melanin levels [75]. *P. vera* L. hull extracts were tested in vitro for their activity against various cancer cells. The findings showed that the Antep flavanone extracts, which contain hesperetin, hesperidin, and naringenin, had an IC_50_ value of 25 ppm, whereas the Antep flavone extracts, which contain eupatorin, apigenin, and luteolin, had an IC_50_ value of 75 ppm against the HeLa cells. When tested against MCF-7 cells, IC_50_ values were highest for Siirt phenolic acid-solid, acid hydrolysis1 (PASA1), Urfa PASA2, and Antep base-hydrolyzed phenolic acids (BHPA) extracts (25, 50, and 60 ppm), as well as for Siirt flavonol, Antep flavonol, and Urfa flavan-3-ol extracts (35, 47, and 60 ppm). The extracts of Siirt, Antep, and Urfa *P. vera* L. PASA1 demonstrated cytotoxic effects on OE-33 cells, as indicated by their IC_50_ values of 40, 45, and 45 ppm [76]. A study conducted in vitro shown that *P. vera* L. ethanolic extracts, at different doses (62.5, 125, 250, 500, and 100 µg/mL), exhibited cytotoxic effects on breast and ovarian cancer cells. The extracts may include bioactive compounds with possible anticancer activities, which could explain their cytotoxic effects [77].

The cytotoxic activity, anti-angiogenesis, and apoptosis induction of four distinct pistachio hull extracts against different cancer cells were evaluated in vitro using several methods, including the trypan blue and MTT assays. Overall, the ethyl acetate extract exhibited varying degrees of toxicity, with liver cancer cells being the most resistant and breast cancer cells being the most vulnerable. Within 72 h, the ethyl acetate extract showed a considerable inhibitory impact on colon cancer cells (IC_50_ = 23.00 and 25.15 µg/mL) and breast cancer cells (IC_50_ = 21.20 µg/mL). The findings also demonstrated that as extract concentrations increased, cell viability decreased (from 45.5% at 25 µg/mL to 4.5% at 400 µg/mL). Bcl-2-associated X protein (Bax) expression was shown to be up-regulated in treated cells, while anti-apoptotic B-cell lymphoma-2 (Bcl-2) expression dropped at doses of 12.5 and 25 µg/mL but was marginally elevated at a dose of 50 µg/mL. The Sub-G1 phase percentage rose from 1% at 0 µg/mL to 25.53% at 25 µg/mL, following a 48 h exposure to extracts. Additionally, there was a notable suppression of angiogenesis by the extracts at the maximum dose (50 µg/mL) [78]. Using both in vitro and in vivo experimental models of breast cancer, the anticancer activity of the most cytotoxic fraction of *P. vera* L. ethyl acetate extraction was examined. F13b1 was determined to be the most cytotoxic fraction of the *P. vera* L. hull extracts. The chemical profile of F13b1 revealed quercetin and gallic acid. It was shown that after 72 h of incubation, cell viability significantly decreased as concentration of treatment increased (from 66.5% at 7.8 µg/mL to 2.02% at 250 µg/mL). The percentage of Sub-G1 phase increased to 62.1% after treatment with the compounds at a high dose (32 µg/mL). Compound treatment produced a decrease in Bcl-2 expression, while increasing the expression of apoptotic genes, including caspase 3/8, Bax, catalase (CAT), and superoxide dismutase (SOD). In mice with cancer, the treatment also suppressed the growth of tumors at dosages of 12.5 and 25 µg/mL [79]. The purpose of an experiment was to ascertain the anti-proliferative activities of pistachio extracts against colon, liver, and breast cancer cells in vitro. The main active ingredients in raw and roasted pistachios were catechin and gentisic acid. With EC_50_ values of 47.84% and 51.49%, respectively, raw pistachios demonstrated anti-proliferative activities against liver and colon cancer cells, while free-form extracts of roasted pistachios showed relatively high anti-proliferative activity against all cancer cells in dose-dependent manners, without cytotoxicity [80]. The goal of one in vitro experiment was to assess the potential effects of pistachios on colon cancer by examining the expression of genes related to detoxification, cell growth inhibition and apoptosis, and anti-genotoxic activity. After 72 h of incubation at a dosage of 2.5%, treatment with fermentation supernatants derived from raw and roasted pistachios inhibited colon cancer growth more effectively (28.7%). Anti-genotoxic activity was also demonstrated by the 2.5% and 5% fermentation supernatants, which decreased the DNA damage caused by hydrogen peroxide (H_2_O_2_) in colon cancer cells. The mRNA levels of CAT, SOD2, and glutathione S-transferase P (GSTP1) gene expression were markedly elevated following treatment with pistachio fermentation supernatants, especially at a dosage of 5%. Additionally, after being treated with 5% fermentation supernatants, caspase-3 activity—a hallmark of advanced apoptosis—was markedly increased [81].

Utilizing female rats with breast tumors produced by dimethyl-benz(a)anthracene (DMBA), an experiment was conducted to examine the anticancer impact of *P. vera* L. leaf ethanolic extract on breast cancer cells. A strong antioxidant activity against DPPH radicals was demonstrated by *P. vera* L., with an IC_50_ value of 72.6 μg/mL. *P. vera* L. demonstrated good selectivity and dose-dependent cytotoxicity against breast cancer cells. The administration of *P. vera* L. leaves dramatically reduced the levels of nitric oxide (NO), malondialdehyde (MDA), carcinoembryonic antigen (CEA), tumor necrosis factor (TNF-α), interleukin (IL-4/6/10/1β), carbohydrate antigen 19-9 (CA19.9), and cancer antigen 15.3 (CA15.3). Furthermore, *P. vera* L. leaves had a strong antioxidant impact on breast cancer cells by significantly raising levels of reduced glutathione (GSH), mammary glutathione peroxidase (GPx), SOD, and CAT [82]. An in vitro experimental investigation was planned to investigate the potential anticancer effects of the liposomal form of pistachio hull extract on HepG2 cells. Up-regulation of p53 and Bax levels and down-regulation of Bcl-2 expression, with a corresponding up-regulation of Bax/Bcl-2 ratio, demonstrated the liposomal extract’s promising ability to induce apoptosis [83]. An experiment was conducted to examine the in vitro anti-tumor activity and underlying mechanisms of pistachio kernel/pericarp extracts, and in combination with cisplatin, in the treatment of prostate cancer. Compared to treatments that used cisplatin alone, the extracts plus cisplatin demonstrated synergistic effects on the reduction of cell proliferation. Following 24, 48, and 72 h of treatment, the IC_50_ values for pistachio kernel extract and cisplatin were 4.141, 2.140, and 0.884 ug/mL, and for pistachio pericarp extract and cisplatin were 2.754, 2.061, and 0.753 ug/mL, respectively. Also, when extracts and cisplatin were administered to prostate cancer cells, the mRNA expression of Bax and p53 increased, while that of Kallikrein-2 (KLK2), nanog homeobox (NANOG), transforming growth factor (TGF), BCL-2, and TNF-α decreased [84]. The in vitro anticancer effect of pistachio hull essential oil was tested on cancer cells from the liver, stomach, and colon. The findings showed that when cancer cells were treated with pistachio hull essential oil, the β-catenin protein and frizzled class receptor 7 (FZD7) were suppressed. The findings also indicated that the genes for T cell factor 1 (TCF1), catenin beta-1 (CTNNB1), lymphoid enhancer-binding factor 1 (LEF1), and proto-oncogene wnt-1 were down-regulated, whereas glycogen synthase kinase-3 beta (2SK-3β) was up-regulated [85]. An in vitro experiment was conducted to investigate the anticancer effects of several extracts from the green hull of pistachios on colon cancer cells. Fifteen phenolic compounds and fatty acids, including gallic acid, catechin hydrate, 9-octadecenoic acid, 9,12-octadecadienoic acid, and hexadecenoic acid, were found in the n-hexane fraction. Colon cancer cells were more cytotoxically affected by the n-hexane fraction. According to oxidative and cell cycle assessments, the n-hexane fraction increased apoptosis and DNA damage through oxidative stress, which stopped the cell cycle at the sub-G1 phase [86].

Table 4 presents the main results pertaining to *P. vera*’s cytotoxic and anticancer activities.

## 5. Anticancer and Cytotoxic Activities of *Pistacia terebinthus*

Only a few experimental studies have documented *P. terebinthus* L. extracts being examined for their anticancer and cytotoxic activities against different forms of cancer. The essential oil compounds from *P. terebinthus* L. had a dose-dependent cytotoxic activity on breast, ovarian, and colon cancer cells in an in vitro experiment; in most cases, the IC_50_ values were less than 500 µg/mL [72]. The purpose of another in vitro experiment was to assess the potential impact of *P. terebinthus* L. resin extract on the signaling pathways for apoptosis and cytotoxicity in breast cancer cells. The findings showed that even at low concentrations, *P. terebinthus* L. resin exhibited cytotoxic effects. About 50% of the cells died during the 24 h incubation period, when the extract’s IC_50_ dose was determined to be 56.54 μg/mL. There was a noticeable decrease in the quantity of viable cells at extract concentrations of 200, 300, and 500 μg/mL, which were higher than the toxic dose. The cells treated with 100 μg/mL extract had a considerably higher level of caspase-3 protein expression [87]. An in vitro experimental investigation was conducted to determine the anticancer activity and define the chemical composition of the essential oil of *P. terebinthus* L. fruits. The analysis revealed that the main constituents of *P. terebinthus* L. essential oil were β-phellandrene, hexadecenoic acid, pyrogallol, α-humulene, limonene piperitone, cis-11, ethyl stearate, linoleic acid, isopropyl palmitate, ethyl palmitate, ethyl nonadecanoate, 14-sicosadienoic acid, ethyl palmitate, ethyl palmitate, ethyl nonadecanoate, ethyl nonadecanoate, ethyl nonadecanoate, ò-phellandrene, hexadecenoic acid, pyrogallol, α-humulene, and 11-eicosenoic acid. *P. terebinthus* L. may have cytotoxic properties because these compounds are included in its essential oils. *P. terebinthus* L. inhibited lung cancer cell proliferation in a dose-dependent manner. Lung cancer cells treated with *P. terebinthus* L. for 24 h showed cell viability varying from 99.81% to 16.13% for concentrations ranging from 0.5 to 500 μg/mL, with an IC_50_ value of 123.8 μg/mL [88]. A different in vitro experiment examined the cytotoxic efficacy of the essential oils derived from *P. palestina* L. against a number of cancer cells. This oil’s main components were limonene and sabinene. The proliferation of renal and melanoma skin cancer cells was inhibited by these oils, which shown considerable cytotoxic action (IC_50_ values of 204.70 and 356.98 μg/mL, respectively) [89]. The cytotoxic and anticancer effects of *P. palaestina* L. essential oils on colon cancer cells were the goal of an in vitro experiment. The primary components of essential oils were found to be monoterpene hydrocarbons. The findings demonstrated that *P. palaestina* L. oil, at a concentration of 43 μg/mL, considerably decreased the viability of colon cancer cells after 24 and 48 h of treatment and enhanced the anticancer/cytotoxic effects of 5-fluorouracil, a conventional chemotherapy. Following fruit oil treatment, cancer cells showed an anti-migratory impact. *P. palaestina* L. dramatically decreased levels of the chemokine C-X-C motif ligand 8 (CXCL8), which is involved in the growth of cancer cells [90].

The main findings of *P. terebinthus*’s cytotoxic and anticancer activities are shown in Table 5.

## 6. Anticancer and Cytotoxic Activities of *Pistacia khinjuk*

A small number of experimental investigations have reported that *P. khinjuk* extracts were tested for their anticancer and cytotoxic activities against a range of cancer types. In an in vitro experimental investigation, the cytotoxic effects of the essential oil derived from *P. khinjuk* on prostate and breast cancer cells were assessed. After 48 h of treatment, the results showed that the essential oil was toxic, with the IC_50_ values for breast and prostate cancer cells being 29.6 and 37.3–41.1 μg/mL, respectively [28]. The cytotoxic activity of *P. khinjuk* regenerated components was evaluated in vitro and in vivo (in male and female cells) against breast and colon cancer cells. The outcome revealed cytotoxic effects on cancerous cells. In vivo female root extracts were more cytotoxic to breast (IC_50_ = 31.86 μg/mL) and colon (IC_50_ = 59.60 μg/mL) cancer cells than stem and leaf extracts [91]. The cytotoxic effects of an ethanol extract derived from *P. khinjuk* leaves on a number of cancer cells were examined in vitro. The leaves were found to contain a total of 18 distinct flavonoids and phenolic acids. Myricetin was identified as the principal free-flavonoidal aglycone. The extract exhibited moderate cytotoxic activity against all cancer cells, with the IC_50_ values for prostate, liver, breast, and lung cancer cells being 25.6, 98.1, 29.4, and 48.1, respectively [92]. An in vitro experiment was conducted to investigate the possible anti-prostate cancer and cytotoxic effects of copper nanoparticles (CuNPs) infused with *P. khinjuk* leaf extract. The findings demonstrated that while CuNPs had minimal toxicity to normal cells, they showed cytotoxic activity and exceptional effectiveness in suppressing prostate cancer cells. The most effective anti-prostate cancer actions of CuNPs were seen in the LNCaP clone FGC cells. CuNPs had IC_50_ values of 322, 365, 247, and 273 for NCI-H660, DU145, LNCaP clone FGC, and LNCaP clone FGC-Luc2 cells, respectively [93]. The cytotoxic effects of *P. khinjuk* seed extracts on mammary cancer were assessed in vitro. Both aqueous and methanolic seed extracts of *P. khinjuk* exhibited cytotoxic effects at high doses. Starting at 78.125 μg/mL and reaching its maximum concentration of 10,000 μg/mL, the aqueous extract demonstrated a significant reduction in cancer cell proliferation within 24 h of treatment. Throughout the course of treatment, the methanolic extract significantly increased cell proliferation, especially at the highest concentrations of 5000 μg/mL and 10,000 μg/mL [94].

Table 6 displays the main findings of *P. khinjuk*’s cytotoxic and anticancer activities.

## 7. Summary of Anticancer and Cytotoxic Activities of *Pistacia* Species

The results of this review showed that *pistacia* species from various plant parts have anticancer properties. The bioactive compounds that were isolated from *P. vera* L. and *P. terebinthus* L. specifically demonstrated anticancer action against cancer cells found in the breast, colon, liver, stomach, prostate, skin, and lungs. Table 7 provides an evaluation of these compounds and their anticancer potential in *pistacia* species.

The findings also demonstrated that several phenolic compounds from *pistacia* species exhibited strong cytotoxic effects on cancer cells in the lungs, esophagus, colon, ovaries, cervix, prostate, kidneys, and breast. An analysis of phenolic compounds and their cytotoxic potential in *pistacia* species is shown in Table 8.

## 8. Limitations

There are few in vivo mouse models, and most studies were conducted on in vitro models. Although *pistacia* species and their phenolic compounds have anticancer effects on cancer cells, clinical trials have not examined these effects. *Pistacia* species exhibit cytotoxic and anticancer effects on cancer cells; however, the possible mechanisms underlying these activities in most studies are unknown and have not yet been explored. The bioavailability of *pistacia* species and their phenolic compounds has not been sufficiently investigated in any previous study. The fact that each study had a distinct dosage schedule for treatment raises concerns about the validity of the findings. It is also as yet uncertain what the best, most efficient dosage is for treatment. Differences in phenolic component extraction throughout the summarized studies made it challenging to evaluate findings across studies on the effects on cancer cells. Some studies failed to identify or extract the phenolic components from *P. vera* L. and *P. kinjuk* for their anticancer effects. Despite the fact that most studies demonstrated that *pistacia* species and their phenolic compounds were highly toxic to cancer cells, they were beneficial in reducing cell proliferation by promoting cell cycle arrest and apoptosis.

## 9. Conclusions

The review findings indicate that total phenols, flavonoids, anthocyanins, proantocyanidins, gallic acid derivatives, phenolic acids, fatty acids, essential oils, and sterols were the primary phenolic components that were extracted from *pistacia* species using various solvents and extraction techniques.

*P. vera* L. extracts and isolated compounds from various plant parts have been shown to exhibit cytotoxic and anticancer activities against a range of cancer types. Using acetonitrile, methanol, formic acid, and ethyl acetate solvents, essential oils demonstrated cytotoxic activity against cancer cells from the breast, colon, and ovaries. Aqueous extracts of pistachios did not cytotoxically affect liver cancer cells, whereas hexane-based extracts induced toxicity and elevated p53 expression and caspase3 activity. At high dosages, the aqueous/methanolic extracts showed anti-melanogenic and toxic effects on melanoma cells. Phenolic compounds that were extracted using various solvents (n-hexane, methanol, ethanol, ethyl ether, and water) had cytotoxic effects on the adrenal cortex, breast, esophagus, and cervical region. Both ovarian and breast cancer cells showed cytotoxic effects and fewer viable cells when exposed to the ethanolic extracts. Cell viability and angiogenesis decreased in ethyl acetate extract-treated breast cancer cells, whereas apoptosis/cell cycle arrest increased as a result of down-regulating Bcl-2 expression and up-regulating Bax, SOD, CAT, and caspase 3/8 expression. Without being cytotoxic, free-form extracts of roasted pistachios demonstrated strong anti-proliferative activity against breast, colon, and liver cancer cells. When colon cancer cells were treated with fermentation supernatants derived from raw and roasted pistachios, they displayed increased caspase-3 activity, decreased H_2_O_2_-induced DNA damage, and increased CAT, SOD2, and GSTP1 gene expression mRNA levels. Cisplatin and methanolic extracts demonstrated synergistic effects on prostate cancer cells, causing them to undergo apoptosis and lowering their growth. The n-hexane fraction halted the cell cycle at the sub-G1 phase and accelerated apoptosis and DNA damage, although it had a highly toxic effect on colon cancer cells.

Extracts from *P. terebinthus* L. showed cytotoxic and anticancer activities against various cancer types. Essential oils have demonstrated a significant level of cytotoxic effect against cancer cells in the breast, colon, and ovaries. The growth of lung cancer cells was inhibited by essential oils extracted with methanol and ethyl acetate solvents, and these oils may be cytotoxic to these cells. *P. palaestina* L. oils were shown to have cytotoxic effect by preventing the growth of skin cancer cells and kidney cancer cells. Moreover, *P. palaestina* L. oil decreased the migration and viability of colon cancer cells and enhanced the toxic effects of the conventional treatment 5-fluorouracil. *P. terebinthus* L. resin boosted the production of caspase-3 in breast cancer cells treated with methanolic extracts, although it also had toxic effects at low dosages.

A variety of cancer types have been shown to be susceptible to the cytotoxic and anticancer effects of *P. khinjuk* extracts. Prostate and breast cancer cells have been demonstrated to be cytotoxically affected by essential oils. Stem and leaf extracts were less cytotoxic to breast and colon cancer cells than root extracts with ethanol as a solvent. The extracts utilizing petroleum ether and ethanol showed moderate cytotoxic efficacy against lung, liver, prostate, and breast cancer cells. Both the aqueous and methanolic seed extracts showed cytotoxic activities. A significant reduction in cancer cell proliferation was observed in the aqueous extract, whereas the methanolic extract significantly increased it, especially at the highest concentrations.

## 10. Future Directions

This review has demonstrated the anticancer activities of *pistacia* species, which may help in the creation of novel anticancer treatments. The results show that some phenolic compounds from *pistacia* species exhibit anticancer activities; however, the level of toxicity may differ depending on the dosage and usage period. Further experiments are required to discover the optimal dosage and mode of administration for *pistacia* extracts. Additional experiments on possible toxicity at suitable dosages are also needed to make sure the treatment has no negative side effects.

More in vivo experimental models are needed to assess the anticancer and cytotoxic effects of *pistacia* species on cancer cells. In vivo and in vitro investigations are needed to further elucidate the potential mechanisms of the cytotoxic and anticancer actions of *pistacia* species and their phenolic compounds on cancer cells. Additional experiments that concentrate on maximizing the bioavailability of *Pistacia* extracts are required in order to assess their anticancer activities. Clinical studies are required to strengthen the findings that *pistacia* species and their phenolic compounds have therapeutic potential in cancer cells. 

## Figures and Tables

**Table 1:** Phenolic compounds identified in various parts of *P. vera* L.

*Pistacia* Parts	Phenolic Compounds	Quantitative Values	Solvents/Extraction Techniques	Ref.
Kernels	Total phenols, flavonoids, proantocyanidins	Phenols: 128.14–156.42.mg.gallic acid.equivalents/g.dry.weight; Flavonoids: 93.17–130.94.mg.quercetin.equivalents/g.dry.weight; Proantocyanidins: 118.87–151.90.mg.catechin.equivalents/g.dry.weight	Methanol/Ultraviolet–visible spectrophotometer	[44]
Kernels, hulls	Total phenols, flavonoids, proantocyanidins	Kernels, Phenols: 0.94–113.21.mg.gallic acid.equivalents/g.dry.weight; Flavonoids: 0.43–87.03.mg.quercetin.equivalents/g.dry.weight; Proantocyanidins: 0.81–110.60.mg.catechin.equivalents/g.dry.weight Hulls, Phenols: 25.39–169.53.mg.gallic acid.equivalents/g.dry.weight; Flavonoids: 20.85–139.47.mg.quercetin.equivalents/g.dry.weight; Proantocyanidins: 23.80–150.32.mg.catechin equivalents/g.dry.weight	Ultrapure water, methanol and ethanol, acetone, ethyl acetate, n-hexane/Ultraviolet–visible spectrophotometer	[45]
Hulls	Total phenolic and flavonol content, phenolic acids	Total phenolic: 40.6–68.1.mg.tannic acid equivalent/g.dry.weight; total flavonol: 4.01–10.93.mg.quercetin equivalents/g.dry.weight; cyanidin-3-*O*-galactoside: 120.81–181.94.mg/100 g.dry.weight; gallic acid: 27.89–45.25.mg/100.g.dry.weight; catechin: 7.2–11.01.mg/100.g.dry.weight; eriodictyol-7-*O*-glucoside: 7.23–16.02.mg/100.g.dry.weight	Methanol/Colorimetric aluminum chloride	[46]
Skins	Total phenolic, flavonoids and anthocyanins content	Total phenolic: 16.26–17.4.mg/g.dry.weight; total flavonoids: 4.2–4.63.mg/g.dry.weight; total anthocyanins: 8.6–10.2.mg/g.dry.weight	Acetone, ethanol, methanol, water/Ultrasound-assisted and maceration extraction	[47]
Hulls	Total phenols and flavonoids content	Total phenols: 120.31.mg gallic acid/g.dry.weight; total flavonoids: 34.54.mg.catechin equivalent/g.dry.weight	Water/Optimized membrane condition	[48]
Hulls	Flavonol and gallic acid derivatives	Flavonols: 4.37–5.65.g/kg.dry.matter of hulls; gallic acid: 22.2.g/kg.dry.matter of hulls; total gallotannin: 33.g/kg.dry.matter of hulls; penta-*O*-galloyl-*β*-D-glucose: 9.77.g/kg.dry.matter of hulls	Methanol, water, formic acid/Subcritical water and ultrasound-assisted extraction	[49]
Hulls	Total phenolic and flavonoid contents, gallic acid, quercetin	Total phenolic: 23.3 and 14.7.mg.gallic acid/g.dry.weight; total flavonoid: 5.0 and 2.9.mg.quercetin/g.dry.weight; gallic acid: 1.9 and 1.5.mg/g.dry.weight; quercetin: 0.025 and 0.009.mg/g.dry.weight	Ethanol, water/Microwave-assisted extraction	[50]
Seeds	Phenolic acids	Gallic acid: 122–225.77.µg/g; catechin: 5.96–25.21.µg/g; diosmin: 22.60.µg/g; epicatechin: 78.20.µg/g; luteolin: 12.97.µg/g; rosmarinic acid: 4.32.µg/g; sinapic acid: 39.14.µg/g; syringaldehyde: 1.85–15.12.µg/g; syringic acid: 12.60.µg/g; vanillin: 1.06–3.22.µg/g	Ethanol, water/Microwave-assisted extraction	[51]
Leaves	Total phenolic and flavonoid contents	Total phenolic: 1.60–196.35.mg.gallic acid/g.dry.weight; total flavonoid: 1.15–83.34.mg catechin/g.dry.weight	Methanol/Microwave-assisted and maceration extraction	[52]
Hulls	Phenolic and flavonoid compounds	Anacardic acids: 3197.70.mg/100.g. dried hull; carotenoids: 4.93.mg/100.g.dried.hull; phytosterols: 192.22.mg/100.g.dried.hull; fatty acids: 1500.12.mg/100.g.dried.hull; quercetin-3-*O*-glucoside: 6.27.mg/g; quercetin, myricetin, and luteolin: 5.53.mg/g	Methanol/Gas chromatography–mass spectrometry	[53]
Kernels	Gallic acid, caffeoylquinic acid, anacardic acid, genistein 7-*O*-glucoside, isoquercetin, quercetin galloyl hexoside, kaempferol hexoside, naringenin, procyanidin dimer	NA	Ethanol, water/Subcritical fluid extraction	[54]
Skins	Total phenolic and flavonoid contents/compounds	Total phenolic: 360 to 463.mg.gallic acid/100.g.dry.weight; total flavonoid: 20.6.mg.quercetin/100.g.dry.weight; gallic acid: 75.µg/g.dry.weight; procyanidin dimer: 55.µg/g.dry.weight; (+)-catechin: 140.µg/g.dry.weight; (-)-epicatechin: 27.53.µg/g.dry.weight; quercetin: 13.7.µg/g.dry.weight; quercetin-*O*-hexoside: 2.68.µg/g.dry.weight; isoquercitrin: 49.3.µg/g.dry.weight; myricetin: 1.6.µg/g.dry.weight; eriodictyol: 13.7.µg/g.dry.weight; naringenin: 1.9.µg/g.dry.weight; luteolin: 30.4.µg/g.dry.weight; cyanidin-*O*-galactoside: 21.14.µg/g.dry.weight	Acidified methanol/Electrospray ionization, quadrupoletime of flight mass spectrometry	[55]
Oils, flours	Fatty acids, phenolic and flavonoid compounds	Pistachio oils-oleic acid: 53.5–55.3; linoleic acid: 29–31.4; carotenoids: 48–56.µg/g.oil Pistachio flours-gallic acid: 23–36.µg/g.flour.dry.weight; (+)-catechin: 38–65.6.µg/g.flour.dry.weight; cyanidin-3-*O*-galactoside: 21–23.µg/g.flour.dry.weight	Acidified methanol/Screw-press operations	[56]
Kernels	Flavonoid compounds	Proanthocyanidins: 268.12.mg/100.g.edible nuts; *trans*-resveratrol isoflavones: 12.mg/100.g.edible nuts; daidzein: 3.68.mg/100.g.edible nuts; genistein: 3.40.mg/100.g.edible nuts	Methanol/Hydrophilic extraction	[57]
Shells	Total phenols and flavonoids	Total phenols: methanol extraction: 381.mg.gallic acid/g.dry.weight, ethanol extraction 293.mg.gallic acid/g.dry.weight; total flavonoids: methanol extraction: 359.mg.catechin/g.dry.weight, ethanol extraction 283.mg.catechin/g.dry.weight	Methanol, ethanol, water/Microwave-assisted extraction	[58]
Skins, seeds	Phenolic and flavonoid compounds	Seeds: gallic acid: 12.66.µg/g.fresh.weight; catechin: 2.41.µg/g.fresh.weight; eriodictyol-7-*O*-glucoside: 31.91.µg/g.fresh.weight; genistein-7-*O*-glucoside: 47.02.µg/g.fresh.weight; naringenin-7-*O*-neohesperidoside: 37.11.µg/g.fresh.weight; quercetin-3-*O*-rutinoside: 98.08.µg/g.fresh.weight; genistein: 69.15.µg/g.fresh.weight; eriodictyol: 9.37.µg/g.fresh.weight; daidzein: 42.45.µg/g.fresh.weight; apigenin: 0.59.µg/g.fresh.weight Skins: gallic acid: 1453.31.µg/g.fresh.weight; catechin: 377.45.µg/g.fresh.weight; epicatechin: 104.8.µg/g.fresh.weight; eriodictyol-7-*O*-glucoside: 365.68.µg/g.fresh.weight; naringenin-7-*O*-neohesperidoside: 118.82.µg/g.fresh.weight; quercetin-3-O-rutinoside: 5.05.µg/g.fresh.weight; eriodictyol: 63.17.µg/g.fresh.weight; quercetin: 17.75.µg/g.fresh.weight; naringenin: 11.44.µg/g.fresh.weight; luteolin: 18.97.µg/g.fresh.weight; kaempferol: 0.95.µg/g.fresh.weight; cyanidin-3-*O*-galactoside: 5865.12.µg/g.fresh.weight; cyanidin-3-*O*-glucoside: 32.56.µg/g.fresh.weight	Methanol, water, n-hexane/Colorimetric assay, rotary evaporation, ultraviolet–visible spectrophotometer	[59]
Virgin oils, residual cakes	Flavanols, flavonols, flavanones, anthocyanins, gallotannins	Flavanols: 4.6–52.mg/kg.fresh.weight; flavonols: 16–130.mg/kg.fresh.weight; flavanones: 12–71.3.mg/kg.fresh.weight; anthocyanins: 83–218.mg/kg.fresh.weight; gallotannins: 4–46.mg/kg.fresh.weight	Methanol, water, n-hexane/Electrospray ionization, tandem mass spectrometry, diode array detection	[60]
Kernels	Hydroxybenzoic acids, flavanols, flavones, flavan-3-ols, flavanones flavanonols	Hydroxybenzoic acids: 116–422.µg/100.g; flavanols: 19–137.µg/100.g; flavones: 0.44–3.18.µg/100.g; flavanones: 1.05–8.31.µg/100.g; flavanonols: 0.44–1.23.µg/100.g	Methanol, acidified water, formic acid/Ultra-high-performance liquid chromatography high resolution mass spectrometry	[61]

NA: not available.

**Table 2:** Phenolic compounds identified in various parts of *P. terebinthus* L.

*Pistacia* Parts	Phenolic Compounds	Quantitative Values	Solvents/Extraction Techniques	Ref.
Fruits	Total phenolic and flavonoid contents, fatty acids	Total phenolic: 11.01–57.07.µg.pyrocatechol·mg^−1^; total flavonoid: 19.18–60.33.µg.quercetin·mg^−1^ oleic acid: 52.5%; palmitic acid: 21.6%; linoleic acid: 19.1%	Ethanol, water, dichloromethane, n-hexane/Gas chromatography–flame ionisation detection	[20]
Leaves	Essential oils	α-pinene: 19.9%; β-pinene: 8.5%; sabinene: 15.4%; limonene: 4.9%; o-cymene: 4.7%; β-phellandrene: 3.2%	n-hexane/Ultra-gas chromatograph, gas chromatography–flame ionization	[21]
Fruits	Fatty acids	Oleic acid: 52.3%; linoleic acid: 19.7%; palmitic acid: 21.3%	Diethyl ether, ethanolic potassium hydroxide, petroleum ether, chloroform/Clevengertype apparatus	[62]
Fruits	Fatty acids, sterols	Oleic acid: 46.9%; linoleic acid: 21.7%; palmitic acid: 21.6%; *β*-sitost: 1268.5.mg/kg; 5-aven: 56.7.mg/kg; 5.24-stigma: 24.5.mg/kg	Petroleum ether, ethanolic potassium hydroxide/Gas chromatography–mass spectrometry	[63]
Fruits	Fatty acids	Oleic acid: 34.8%; linoleic acid: 17.3%; palmitic acid: 21.7%	Petroleum ether/Gas chromatography–mass spectrometry, rotary evaporation	[64]
Fruits	Total phenolic and flavonoid contents, phenolic acids	Total phenolic: 70.2–222.6.mg.garlic acid/100.g; total flavonoid: 164–389.2.mg.quercetin/100.g; gallic acid: 9.8–16.9.mg/100.g; 3,4-Dihydroxybenzoic acid: 0.8–2.2.mg/100.g; catechin: 0.8–1.6.mg/100.g	Methanol, water, petroleum ether/Liquid and gas chromatography	[65]
Fruits	Total phenolic, flavonoid, and oil contents, phenolic compounds, fatty acids	Total phenolic: 213.4–251.2.mg gallic acid/100.g; total flavonoid: 3371.1–3413.7.mg.catechin/100.g; total oil: 40.4–42.6%; quercetin: 330.9–467.7.mg/100.g; kaempferol: 2.69–4.17.mg/100.g; naringenin: 3.75–4.08.mg/100.g; gallic acid: 7.8–14.7.mg/100.g; (+)-Catechin: 15–21.3.mg/100.g; protocatechuic acid: 7.9–9.2.mg/100.g; 1,2-Dihydroxybenzene: 4.1–11.7.mg/100.g; rutin trihydrate: 1.9–7.2.mg/100.g; oleic acid: 48–49.1%; linoleic acid: 22.2–23.4%; palmitic acid: 22.1–23.6%	Methanol, n-hexane, soxhlet and petroleum benzene/Spectrophotometer, rotary vacuum evaporator, gas chromatography, flame ionization detector	[66]
Fruits	Total carotenoid and phenolic contents, fatty acids	Total carotenoid: 58.3–68.2.mg/kg.oil; total phenolic: 10.5–19.2.µg.gallic acid.equivalent; lutein: 10.9–14.1.mg/kg.oil; β-Carotene: 6–8.3.mg/kg.oil; oleic acid: 46.6–47.5%; linoleic acid: 22.1–22.5%; palmitic acid: 22.5–23%	Methanol, n-hexane/Gas chromatography, shimadzu prominence high-performance liquid chromatography	[67]
Fruits	Total phenolic and flavonoid contents	Total phenolic- methanol extract: 122.7.µg.pyrocatechol.equivalents/mg, acetone extract: 61.µg.pyrocatechol equivalents/mg; total flavonoid- methanol extract: 22.6.µg.quercetin equivalents/mg, acetone extract: 5.4.µg.quercetin equivalents/mg	Methanol, acetone/Soxhlet apparatus, ultraviolet spectra	[68]
Fruits	Total phenol and flavonoid contents, fatty acids, essential oils	Total phenol-methanol extract: 241.µg.gallic acid equivalents/1.g, ethyl acetate extract: 237.1.µg.gallic acid equivalents/1.g; total flavonoid- methanol extract: 47.µg.quercetin equivalents/1.g, ethyl acetate extract: 112.3.µg.quercetin equivalents/1.g; oleic acid: 53.6%; palmitic acid: 25.6%; linoleic acid: 19%; α-pinene: 26.3%; *trans*-β-ocimene: 15.8%; _D,L_-limonene: 14%	Methanol, ethyl acetate/Soxhlet apparatus, ultraviolet–visible double beam spectrophotometer, gas chromatography–mass spectrometry, aluminium chloride colorimetric	[69]
Leaves	Total phenolic content	Total phenolic- methanol extract: 18.3.g.gallic acid.equivalents/100.g, ethanol extract: 17.1.g.gallic acid.equivalents/100.g, acetone extract: 19.3.g.gallic acid.equivalents/100.g	Methanol, ethanol, acetate/Folin–Ciocalteu assay	[22]

**Table 3:** Phenolic compounds identified in various parts of *P. khinjuk*.

*Pistacia* Parts	Phenolic Compounds	Quantitative Values	Solvents/Extraction Techniques	Ref.
Seeds	Total phenolic and flavonoid contents, oleic acid, palmitic acid, linoleic acid	Total phenolic: 11.01–57.07.µg.pyrocatechol·mg^−1^; total flavonoid: 19.18–60.33.µg.quercetin·mg^−1^ oleic acid: 59.4%; palmitic acid: 9.5%; linoleic acid: 27.5%	Ethanol, water, dichloromethane, n-hexane/Gas chromatography–flame ionisation detection	[20]
Fruits	Essential oils	Phellandrene: 52.33%; α-pinene: 15.27%; octadecanoic acid: 6.26%; Δ-limonene: 4.08%	Water/Gas chromatography–mass spectrometry	[70]
Hulls, kernels	Fatty acids	Hulls, oleic acid: 63.55%; palmitic acid: 19.44%; linoleic acid: 13.57% Kernels, oleic acid: 61.11%, linoleic acid: 20.09%; palmitic acid: 16.11%) behenic acid: 0.94%; lauric acid: 0.22%; myristic acid: 0.20%; arachidic acid: 0.18%	n-hexane/ Gas–liquid chromatography	[24]
Hulls, kernels	Phenolic compounds, fatty acids	Hulls, phenol: 19.mg g^−1^ dry.weight; flavonol: 22.mg g^−1^ dry.weight; flavonoid: 6100.µg^−1^ dry.weight; anthocyanin: 490.µg^−1^ dry.weight; oleic acid: 22.8–54.1%; linoleic acid: 7.2–57.5% Kernels, phenol: 3.mg g^−1^ dry.weight; flavonol: 1.mg g^−1^ dry.weight; flavonoid: 500.µg^−1^ dry.weight; anthocyanin: 30.µg^−1^ dry.weight; oleic acid: 18.5–34.5%; linoleic acid: 3.2–13.4%	Methanol/Gas chromatography–flame ionisation detection	[25]
Leaves	Essential oils	Myrcene: 18.7%; α-eudesmol: 12.3%; β-eudesmol: 9.3%; 1,7-di-epi-β-cedrene: 7.3%; bicyclogermacrene: 5.6%; δ-eudesmol: 4.9%	Water/Gas chromatography–mass spectrometry	[71]
Hulls, kernels, shells	Phenolic compounds	Hulls, total phenolic: 25.9.mg.gallic acid/g.dry.mass; total flavonoid: 12.2.mg.catechin/g.dry.mass; caffeic acid: 230.1.μg/g.dry.mass; sinapic acid: 44.2.μg/g.dry.mass; ferulic acid: 42.1.μg/g.dry.mass; vanillic acid: 3.1.μg/g.dry.mass; *p*-hydroxybenzoic acid: 20.6.μg/g.dry.mass; syringic acid: 11.7.μg/g.dry.mass; rutin: 1.9.μg/g.dry.mass Kernels, total phenolic: 3.1.mg.gallic acid/g.dry.mass; total flavonoid: 1.4.mg.catechin/g.dry.mass; caffeic acid: 17.3.μg/g.dry.mass; sinapic acid: 11.4.μg/g.dry.mass; *p*-coumaric acid: 13.2.μg/g.dry.mass; syringic acid: 7.3.μg/g.dry.mass Shells, total phenolic: 4.1.mg.gallic acid/g.dry.mass; total flavonoid: 2.7.mg.catechin/g.dry.mass; caffeic acid: 95.4.μg/g.dry.mass; sinapic acid: 16.7.μg/g.dry.mass; ferulic acid: 27.2.μg/g.dry.mass; vanillic acid: 5.6.μg/g.dry.mass; *p*-hydroxybenzoic acid: 21.4.μg/g.dry.mass	Methanol/Folin–Ciocalteu colorimetric, colorimetric assay	[27]
Hulls	Essential oils	β-Caryophyllene: 25.3%; myrcene: 16.5%; α-pinene: 14.9%; limonene: 9.8%; α-humulene: 5.7%	Water/Gas chromatography–mass spectrometry	[28]
Fruits	Total phenolic and flavonoid content	Total phenolic, methanol: 260.7.mg.gallic acid/g.dry.weight; water: 255.6.mg.gallic acid/g.dry.weight; n-hexane: 43.5.mg.gallic acid/g.dry.weight; chloroform: 94.6.mg.gallic acid/g.dry.weight Total flavonoid, methanol: 191.3.mg.quercetin/g.dry.weight; water: 182.8.mg.quercetin/g.dry.weight; n-hexane: 53.8.mg.quercetin/g.dry.weight; chloroform: 84.6.mg.quercetin/g.dry.weight	Methanol, water, n-hexane, chloroform/Phosphomolybdenum, ferric thiocyanate, spectrophotometric	[29]

**Table 4:** Cytotoxic and anticancer activities of *P. vera* L. against various cancer types.

Cancer Types (Cell Lines)	Plant Parts/Active Compounds	Extraction Techniques and Application	Biological Activity (Bioassays)	Outcomes	Ref.
Breast (MCF-7), colon (LoVo), ovarian (2008)	Flowers, branches, leaves, galls, berries, nuts/Essential oils	Acetonitrile, methanol, formic acid, and ethyl acetate were employed as solvents. Plant parts were hydrodistilled for four hours using a Clevenger apparatus. The oils were gathered and kept in brown, airtight vials at 4 °C until they were examined. Following the dissolution of oils in DMSO, the resulting stock solutions were diluted in culture medium and kept at −20 °C. Gas chromatography analysis combined with a mass spectrometer detector was used to identify essential oils	Cytotoxic activity (MTT assay)	Essential oils frequently exhibited cytotoxic activity against all types of cancer cells	[72]
Liver (HepG2)	Kernels	The pistachio sample was pulverized and extracted using two different methods: the oil phase and the precipitate phase. Hexane was used to separate the two phases. The precipitate phase was dissolved in dimethylsulfoxide to expose cells in the media	Cytotoxic activity (MTT assay)	Aflatoxin B1 toxicity, as well as p53 expression and caspase3 activity in HepG2 cells, were all increased by pistachio extract	[73]
Liver (HepG2)	Nuts/Phenolic acids	Weighed *P. vera* L. powders (30 g each) were placed in individual Erlenmeyer flasks with 300 mL of distilled water. The mixes were allowed to cool after being heated to 60 °C for 30 min while being stirred occasionally. After that, they were filtered, and the filtrates were kept in storage at 4 °C. Using the traditional cold maceration procedure, the ethanolic extracts were made. After being weighed, 30 g of dried plant powder was soaked in 150 mL of 96% standard-grade ethanol. In an incubator with constant shaking, the mixes were incubated for 72 h at 37 °C and 120 rpm. The extracts were identified by gas chromatography–mass spectroscopy	Cytotoxic activity (MTT assay)	Aqueous extracts of *P. vera* L. did not cause any cytotoxicity in HepG2 cells	[74]
Melanoma (SKMEL-3)	Hulls/Total phenolic and flavonoid contents	Following the separation and drying of *P. vera* L. hulls at 40 °C, 100 g of dried powder was extracted three times at ambient temperature using a percolation apparatus and 80% aqueous methanol. Once filtered, the mixture of extracts was vacuum-evaporated, freeze-dried, and kept at 4 °C. Using the Folin–Ciocalteau method and the aluminum chloride colorimetric method, respectively, the extracts’ total phenolic and flavonoid contents were ascertained	Cytotoxic activity (MTT assay)	The extracts demonstrated potent cytotoxic and anti-melanogenic activities on melanoma cells when administered at a high dosage	[75]
Breast (MCF-1), esophagus (OE-33), adrenal cortical (ACC-201), cervical (HeLa)	Hulls/Phenolic compounds, phenolic acids, flavanone, flavonol, flavan-3-ol, flavone	Phenolic compounds: *P. vera* L. hulls were semi-darkly dried at 40 °C, freeze-dried, crushed into a powder, extracted with n-hexane in a Soxhlet apparatus for six hours, evaporated using a rotary evaporator system, lyophilized once more, and then stored at −20 °C Phenolic acids: For one hour at 25 °C, *P. vera* L. hulls were extracted twice using 80% methanol 5/1 (*v*/*v*). At low pressure, the extracts were evaporated until they were completely dry. Following the dissolution of half of the dry extract in 12 mL of water with a pH of 2.0, three liquid-liquid extractions using 12 mL of ethyl ether were conducted Flavanones: After being extracted for two hours at 90 °C using 50 mL of 80% ethanol, *P. vera* L. hulls were filtered and vacuum-evaporated until they were completely dry Flavonols: In a Soxhlet apparatus, dried *P. vera* L. hulls were refluxed for one hour with 100 mL of 95% (*v*/*v*) aqueous methanol and 30 mL of 25% (*w*/*w*) hydrochloric acid. Following filtration, *P. vera* L. hulls were extracted twice for ten minutes using 60 mL of methanolic solution and vacuum-dried Flavan-3-ols: The dried *P. vera* L. hulls were extracted using 40 mL of methanol in an ultrasonic bath at 60 °C for two hours, followed by a 15 min centrifugation at 4500 rpm Flavones: For 20 min, dried *P. vera* L. hulls were suspended in 150 mL of ethyl ether at 25 °C	Cytotoxic activity (MTT assay)	The most frequently observed cytotoxic effects of *P. vera* L. hull extracts were against MCF-7, which was followed by OE-33, HeLa, and ACC-201	[76]
Breast (MCF-7), ovarian (A2780)	Fruits/Total phenolic, flavonoid, tannin, alkaloid, carotenoid, and steroid contents	Total phenolics: 200 L of crude extract, 2 mL of distilled water, and 500 L of Folin–Ciocalteu reagent were combined in a vial, and the mixture was pipetted in and out for approximately one minute. Three minutes were then allowed to mix at room temperature Total flavonoids: The crude extract made up 50 mL of the 5 mL volume, along with 300 mL of sodium nitrate, 1 mL of methanol, and 4 mL of distilled water. A further 10 min of incubation was conducted after 5 min, followed by 300 L of 10% aluminum chloride. Ultimately, 2 mL of sodium hydroxide were added, and the remaining capacity was filled to 10 mL with purified water. For 15–20 min, it was left to rest at room temperature Total tannins: By adding distilled water (750 L), the Folin–Ciocalteu reagent (500 L), 35% sodium carbonate (1000 L), 100 L plant extract, and 7650 L distilled water, the total tannin content was determined. Water was used in place of plant extract as a control. The optical density of this mixture was measured at 725 nm after it had been at room temperature for 30 min Total alkaloids: Chloroform extraction was used to assess the total alkaloid content. A separating funnel (1:1 phosphate buffer–bromocresol solution) was used to vigorously mix 1000 L of plant extract after it had been diluted in a few drops of hydrochloric acid (2N). After diluting the combination with chloroform, the optical density was measured at 470 nm Total carotenoids: Using Thaipong’s approach, the total carotenoid content was determined. Absorbance was measured at 470 nm after the extract was dissolved in n-hexane Total steroids: Chloroform and sulfuric acid (10 mL each) were used to dissolve the extracts in order to assess the total steroid content Gas chromatography–mass spectroscopy was performed to identify the extracts	Cytotoxic activity (MTT assay)	*P. vera* L. ethanolic extracts reduced the number of viable cells in MCF-7 and A2780 cells. On both cancer cells, however, the extracts demonstrated cytotoxic effects	[77]
Colon (HT-29 and HCT-116), liver (HepG2), lung (H23), breast (MCF-7), cervical (Ca Ski)	Hulls	At room temperature, the red pistachio hulls were dried, ground into a powder, and then extracted using water, methanol, ethyl acetate, and hexane (3 × 2500 mL). A R110 Rotavapor was used to decant and concentrate the extracting solvent at 40 °C, and it was then stored at 4 °C	Cytotoxic activity, viability (MTT assay and trypan blue) Apoptosis/cell cycle arrest (gene expression assay and flow cytometric analysis) Angiogenesis (chick chorioallantoic membrane assay)	Breast cancer cells were the most susceptible to the ethyl acetate extract’s various levels of toxicity, whereas liver cancer cells were the most resistant. Cell viability and angiogenesis declined with increasing amounts of ethyl acetate extract. In treated breast cancer cells, Bcl-2 expression decreased while Bax expression increased	[78]
Breast (MCF-7)	Hulls/Gallic acid, quercetin	The dried and powdered *P. vera* L. hulls were immersed in ethyl acetate. Using filter paper, the extract was separated from the residue, which was then twice more extracted using ethyl acetate solvent. The solvent was removed using a rotary evaporator at 40 °C, producing a dark brown crude extract that was then kept at 4 °C. To find the most cytotoxic fraction out of 14—fraction number 13 (F13)—*P. vera* L. ethyl acetate extract was subjected to column chromatography using a glass column. After that, a glass column chromatography using ethyl acetate and dichloromethane combinations was performed on F13. Following thin-layer chromatography monitoring of the isolated fractions, seven suitable fractions were subsequently mixed and dried. Following analysis of the separated fractions using thin layer chromatography, four suitable fractions were mixed and dried in preparation for the subsequent MTT test. Faction number 1 (F13b1), which contained roughly 10 mg, was ultimately determined to be the most effective fraction or pure compound	Cytotoxic activity, cell viability (MTT assay) Apoptosis/cell cycle arrest (gene expression assay and flow cytometric analysis) Anti-tumor/tumor volume in mice (histopathology observations)	Cell viability dramatically declined as F13b1 concentration increased. Bcl-2 expression decreased as a result of compound treatment, although SOD, Bax, CAT, and caspase 3/8 expression increased. The treatment also inhibited the growth of tumors in animals with cancer	[79]
Liver (HepG2), colon (Caco-2), breast (MDA-MB-231)	Nuts/Total phenolics and flavonoids	After being treated with 80% cooled acetone for 5 min by a Virtis High Speed Homogenizer, 4 g of raw and roasted pistachios was vacuum-filtered, and the acetone was removed using a rotary evaporator set at 45 °C. After adding modest amounts of n-hexane to the methanol-water mixture, the methanol was evaporated, and the remaining residue was diluted with MilliQ water-saving free phenolics at −20 °C. A high-performance liquid chromatography method was used to identify the phytochemical profiles of both raw and roasted pistachios	Cytotoxicity and anti-proliferative activities (methylene blue assay)	Free-form extracts of roasted pistachios showed relatively high anti-proliferative activity against all cancer cells in dose-dependent manners, without cytotoxicity, whereas raw pistachios displayed anti-proliferative activities against liver and colon cancer cells	[80]
Colon (LT97)	Nuts	The experiment involved the use of 2 g of ground pistachios. Following 5 min of α-amylase incubation and 2 h of pepsin incubation at 37 °C, pistachio samples were dialyzed under semi-anaerobic conditions (6 h, 37 °C) using intestinal fluid containing pancreatin and oxgall (26 and 50 mg, respectively) in 5 mL of 11 mM bicarbonate buffer	Genotoxic and anti-genotoxic activities (comet assay) Apoptosis (flow cytometric analysis and caspase assay)	Colon cancer cells treated with fermentation supernatants made from raw and roasted pistachios showed reduced H_2_O_2_-induced DNA damage, elevated CAT, SOD2, and GSTP1 gene expression mRNA levels, and increased caspase-3 activity	[81]
Breast (MCF-7 and MDA)	Leaves/Total phenolic content	*P. vera* L. leaves were tested for phenolic content by dissolving 5 mg of the extract in 10 mL of acetone and water. Then, 0.8 mL of the 7.5% sodium carbonate solution and 1.0 mL of the Folin–Ciocalteu reagent that had been diluted ten times were added	Anti-tumor, antioxidants, anti-inflammatory (ABTS radical scavenging activity)	*P. vera* L. leaves demonstrated intriguing potential as antioxidants and anti-inflammatory agents by focusing on the expression of oxidative stress markers and pro-inflammatory cytokines	[82]
Liver (HepG2)	Hulls	*P. vera* hulls that had been dried and ground up were submerged in methanol. Liposomal preparation was carried out using the thin-film hydration technique	Apoptosis (flow cytometric analysis)	*P. vera* hulls extract in liposomal form showed encouraging potential in causing liver cancer cells to undergo apoptosis	[83]
Prostate (PC-3)	Pericarp, kernel	Using methanol, the pistachio kernel and pericarp were ground up and extracted	Anti-proliferation (MTT assay) Apoptosis (real-time technique)	The extracts and cisplatin showed synergistic effects on prostate cancer cells, reducing their proliferation and inducing their apoptosis	[84]
Colon (CACO_2_), liver (PLC/PRF/5), gastric (AGS)	Hulls/Essential oils	As solvents, methanol and ethyl acetate were used. Using a Clevenger apparatus, *P. vera* L. hulls were hydrodistilled. The oils were collected and stored in sealed containers. Oils were dissolved in DMSO, and the resulting stock solutions were then diluted. To identify essential oils, gas chromatography analysis was employed	Anticancer (MTT assay, real-time technique, western blot analysis, silico analysis)	β-catenin protein and FZD7 were inhibited when pistachio hull essential oil was applied to cancer cells	[85]
Colon (HT-29)	Hulls/Phenolic compounds and fatty acids	Phenolic compounds and fatty acids were extracted from the green pistachio husk using n-hexane, methanol, and water as solvents. Using gas chromatography and liquid chromatography/tandem mass spectrometry analysis, phenolic compounds and fatty acids were identified	Cytotoxic activity (MTT assay) Apoptosis/cell cycle arrest (gene expression assay and flow cytometric analysis)	The n-hexane fraction had a greater cytotoxic effect on colon cancer cells. Through oxidative stress, the n-hexane fraction accelerated apoptosis and DNA damage, halting the cell cycle at the sub-G1 phase	[86]

**Table 5:** Cytotoxic and anticancer activities of *P. terebinthus* L. against various cancer types.

Cancer Types (Cell Lines)	Plant Parts/Active Compounds	Extraction Techniques and Application	Biological Activity and Bioassays	Outcomes	Ref.
Breast (MCF-7), colon (LoVo), ovarian (2008)	Flowers, branches, leaves, galls, berries, nuts/Essential oils	As solvents, acetonitrile, formic acid, methanol, and ethyl acetate were used. A Clevenger apparatus was used to hydrodistribute plant pieces for four hours. Before being analyzed, the oils were gathered and kept in sealed brown vials at 4 °C. In order to create stock solutions, which were kept at −20 °C, oils were first dissolved in DMSO and then diluted in culture medium. By using a mass spectrometer detector in conjunction with gas chromatography analysis, essential oils were identified	Cytotoxic activity (MTT assay)	There was a high frequency of cytotoxic activity from essential oils on MCF-7 cells (41%), LoVo cells (70%), and 2008 cells (71%)	[72]
Breast (MDA-MB-23)	Seeds/Resin	Four extractions at room temperature were performed after the resin was macerated with 80% methanol for 24 h each. Under vacuum, the extracts were mixed and concentrated at 37 °C in a rotating evaporator. After being lyophilized, all extracts were kept at −20 °C. A precision scale was used to weigh 4 mg of the extract, which was then transferred into an Eppendorf tube to create the extract stock	Cytotoxic activity (MTT assay) Apoptosis (Hoechst staining, western blot analysis)	The resin of *P. terebinthus* L. showed cytotoxic effects at low doses. The expression of the caspase-3 protein was significantly increased in breast cancer cells treated with the extracts	[87]
Lung (A549)	Fruits/Essential oils	The solvents used were methanol and ethyl acetate. Following hydrodistillation of *P. terebinthus* L. fruits, the oils were collected and stored. The obtained stock solutions were diluted in culture medium and stored at −20°C after the oils were dissolved in DMSO. The identification of essential oils was carried out using gas chromatography–mass spectroscopy	Cytotoxic, anti-proliferative activity (MTT assay)	*P. terebinthus* L. suppressed the proliferation of lung cancer cells and may have cytotoxic effects on cancer cells	[88]
Breast (MCF-7), prostate (LNCaP), renal (ACHN), skin (C32)	Fruits/Essential oils	Using a Clevenger-type device, 200 g of *P. palestina* L. was hydrodistilled for 3 h. After being dried over anhydrous sodium sulfate to eliminate any remaining moisture, the white-yellow essential oils were kept at 40 °C for storage. Using mass spectrometry and gas chromatography, essential oils were examined	Cytotoxic activity (Sulforhodamine B Dye assay)	The cytotoxic activity of *P. palestina* L. oils was demonstrated by inhibiting the proliferation of renal and melanoma skin cancer cells	[89]
Colon (HCT116)	Fruits/Essential oils	A Clevenger apparatus was used to hydro-distilate 300 g of crushed *P. palaestina* L. fruits for 2 h. Following solid phase micro-extraction, the oils were collected individually, dried over anhydrous sodium sulfate, and then kept in storage at 4 °C. Gas chromatography-mass spectrometry and gas chromatography-flame ionization detector analyses were used to ascertain the essential oils’ composition	Cytotoxic activity MTT assay) Anti-migration and proliferation (wound healing assay, enzyme-linked immunosorbent assay)	*P. palaestina* L. oil increased the cytotoxic effects of 5-fluorouracil, a traditional treatment, and reduced the viability and migration of colon cancer cells	[90]

**Table 6:** Cytotoxic and anticancer activities of *P. khinjuk* against various cancer types.

Cancer Types (Cell Lines)	Plant Parts/Active Compounds	Extraction Techniques and Application	Biological Activity (Bioassays)	Outcomes	Ref.
Breast (MCF-7), prostate (PC-3, DU-145)	Hulls/Essential oils	Clevenger-type equipment was used to hydro-distill air-dried material for 3 h in order to separate the essential oil. Following extraction, the essential oil was dried over anhydrous sodium sulphate and kept at 4 °C. The final product had a yield of 0.5% *v*/*w*. Using gas chromatography–mass spectrometry, essential oil was examined	Cytotoxic activity (MTT assay)	Essential oils were shown to have cytotoxic effects on cancer cells	[28]
Breast (MCF-7), colon (HT-29)	Roots, stems, leaves	In vivo male and female *P. khinjuk* roots, stems, leaves, and seeds were utilized. Before being used for the in vitro investigations, the seeds were placed in dry plastic containers and stored in a refrigerator at 4 °C. The seeds were immersed in a 20% commercial bleach solution for 20 min in order to surface sterilize them. Before being inoculated into the murashige and skoog basal media, the kernels were cleaned three times with sterile distilled water after the seed coats were removed. Surface-sterilized seeds were added to murashige and skoog baseline medium supplemented with 30 g/L sucrose and 100 mg/L ascorbic acid, and then solidified with 5.7 g/L agar to initiate the culture. The plantlets’ components were dried individually and kept at 4 °C following a period of cultivation. After adding 50 mL of ethanol for 3 × 24 h, the dried plant samples were shaken at 260 rpm to extract the ethanol. Filtration and solvent evaporation produced crude extracts. After dissolving these extracts in ethanol, solutions of 4000 mg/L in a volume of 10 mL were created. The final quantities of these solutions were 5 mL after they were diluted to 1000 mg/L	Cytotoxic activity (MTT assay)	Compared to stem and leaf extracts, root extracts were more cytotoxic to breast and colon cancer cells	[91]
Breast (MCF-7), prostate (PC-3), lung (A549), liver (HepG2)	Leaves/Flavonoids, phenolic acids	A Soxhlet device was used to extract 100 g of defatted, air-dried *P. khinjuk* leaves in an exhaustive manner using petroleum ether at 40–60 °C. In order to extract the defatted granules, 95% ethanol was used. The extracts were filtered and dried by evaporating them at 40 °C with a lower pressure. *P. khinjuk* extracts were identified using the hyphenated mass spectrometry method	Cytotoxic activity (Sulforhodamine B dye assay)	Moderate cytotoxic activity was demonstrated by the extract against all cancer cells	[92]
Prostate (DU 145, LNCaP clone FGC-Luc2, LNCaP clone FGC, NCI-H660)	Leaves	The percolation (soaking) method, which involved mixing 0.1 kg of plant powder with 0.8 L of water, was used to dry and extract *P. kinjuk* leaves. A rotary evaporator was used to filter the sample and remove all of the organic solvent after it had been stored at 25 °C for two days. This produced crude solid extracts. 40 mL of the extract and 40 mL of Cu(NO3)_2_·3H_2_O (0.25 M) were then mixed together. After that, the resultant mixture was refluxed for 16 h at 75 °C. As a result, NPs (Cu@ *P. Khinjuk*) formed	Anticancer and cytotoxic activities (MTT assay, trypan blue dye, hemocytometer slide)	CuNPs shown cytotoxic activity and remarkable efficacy in inhibiting prostate cancer cells	[93]
Mammary adenocarcinoma (AMN-3)	Seeds	*P. khinjuk* seeds were separated, dried at ambient temperature, ground into a powder using an electronic grinder, and then frozen at −20 °C. From the ground powder, methanolic and aqueous extracts were made. After that, the crude dry extract was labeled and kept at −20 °C	Anti-proliferative and cytotoxic activities (enzyme-linked immunosorbent assay)	At elevated dosages, *P. khinjuk* seed extracts in both aqueous and methanol demonstrated cytotoxic effects. Particularly at the highest concentrations, the methanolic extract markedly enhanced cancer cell proliferation, while the aqueous extract showed a considerable decrease in cell proliferation	[94]

**Table 7:** An overview of the anticancer activities and bioactive compounds in *pistacia*.

Anticancer Activities	*Pistacia* Species	Bioactive Compounds
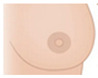 Breast	*P. vera* L.	Gallic acid, quercetin, total phenolics and flavonoids
	*P. terebinthus* L.	Resin
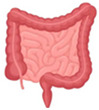 Colon	*P. vera* L.	Total phenolics and flavonoids, essential oils, phenolic compounds and fatty acids
	*P. terebinthus* L.	Essential oils
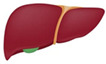 Liver	*P. vera* L.	Total phenolics and flavonoids, essential oils
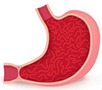 Gastric	*P. vera* L.	Essential oils
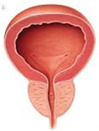 Prostate	*P. terebinthus* L.	Essential oils
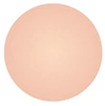 Skin	*P. terebinthus* L.	Essential oils
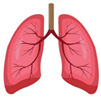 Lung	*P. terebinthus* L.	Essential oils

**Table 8:** A summary of the bioactive compounds and cytotoxic effects of *pistacia*.

Cancer Types	*Pistacia* Species	Bioactive Compounds	Cytotoxic Effects
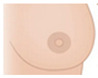 Breast	*P. vera* L.	Essential oils, phenolic and favonoids compounds, tannins, alkaloids, carotenoids, steroids	High
	*P. terebinthus* L.	Essential oils, resin	High
	*P. khinjuk*	Essential oils	High
		Flavonoids, phenolic acids	Moderate
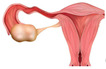 Ovary	*P. vera* L.	Essential oils, phenolic and favonoids compounds, tannins, alkaloids, carotenoids, steroids	High
	*P. terebinthus* L.	Essential oils	High
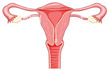 Cervical	*P. vera* L.	Phenolic and favonoids compounds, phenolic acids	High
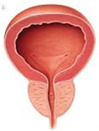 Prostate	*P. terebinthus* L.	Essential oils	High
	*P. khinjuk*	Essential oils	High
		Flavonoids, phenolic acids	Moderate
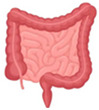 Colon	*P. vera* L.	Phenolic compounds and fatty acids	High
	*P. terebinthus* L.	Essential oils	High
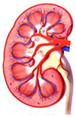 Renal	*P. terebinthus* L.	Essential oils	High
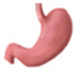 Esophagus	*P. vera* L.	Phenolic and favonoids compounds, phenolic acids	High
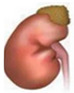 Adrenal cortical	*P. vera* L.	Phenolic and favonoids compounds, phenolic acids	High
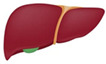 Liver	*P. vera* L.	Total phenolics and flavonoids, phenolic acids	No effect
	*P. khinjuk*	Flavonoids, phenolic acids	Moderate
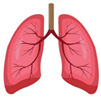 Lung	*P. terebinthus* L.	Essential oils	High
	*P. khinjuk*	Flavonoids, phenolic acids	Moderate
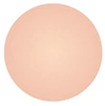 Skin	*P. vera* L.	Total phenolics and flavonoids	High

## Data Availability

Not applicable.

## Abbreviations

2SK-3β	Glycogen synthase kinase-3 beta
Bax	Bcl-2-associated X protein
Bcl-2	B-cell lymphoma-2
BHPA	Antep-base-hydrolyzed phenolic acids
CA15.3	Cancer antigen 15.3
CA19.9	Carbohydrate antigen 19-9
CAT	Catalase
CEA	Carcinoembryonic antigen
CTNNB1	Catenin beta-1
CuNPs	Copper nanoparticles
CXCL8	Chemokine C-X-C motif ligand 8
DMBA	Dimethyl-benz(a)anthracene
FZD7	Frizzled class receptor
GPx	Glutathione peroxidase
GSH	Reduced glutathione
GSTP1	Glutathione S-transferase P
H2O2	Hydrogen peroxide
IC50	Half-maximal inhibitory concentration
IL	Interleukin
KLK2	Kallikrein-2
LEF1	Lymphoid enhancer-binding factor 1
MDA	Malondialdehyde
MTT	3-(4,5-dimethylthiazol-2-yl)-2,5-diphenyltetrazolium bromide
NANOG	Nanog homeobox
NO	Nitric oxide
PASA	Acid hydrolysis
SOD	Superoxide dismutase
TCF1	T cell factor 1
TGF	Transforming growth factor
TNF-α	Tumor necrosis factor
